# Analytical and clinical evaluation of a novel real-time PCR-based detection kit for Mpox virus

**DOI:** 10.1007/s00430-024-00800-4

**Published:** 2024-08-05

**Authors:** Till Bunse, Anne Ziel, Philipp Hagen, George Rigopoulos, Umit Yasar, Hakan Inan, Gurbet Köse, Ulrich Eigner, Rolf Kaiser, Nils Bardeck, Jasmin Köffer, Melissa Kolb, Xiaomei Ren, Deyong Tan, Lizhong Dai, Ulrike Protzer, Jochen M. Wettengel

**Affiliations:** 1 School of Medicine and Health, Institute of Virology, Technical University of Munich, 81675 Munich, Germany; 2 Institute of Virology, Helmholtz Munich, 81675 Munich, Germany; 3 German Center for Infection Research (DZIF), Munich Partner Site, 81675 Munich, Germany; 4 Requalite GmbH, 82166 Graefelfing, Germany; 5 Institute of Virology, University of Cologne, 50935 Cologne, Germany; 6 MVZ Labor Dr. Limbach, 69126 Heidelberg, Germany; 7 National Genetic Detection Technology Application Demonstration Center, Sansure Biotech Inc, Changsha, 410205 People’s Republic of China; 8 Department of Clinical Pharmacology, Xiangya Hospital, Central South University, Changsha, 410008 People’s Republic of China

**Keywords:** Mpox, Clinical diagnostics, Mpox detection assay, MPXV

## Abstract

Outbreaks of emerging diseases, like Mpox in 2022, pose unprecedented challenges to global healthcare systems. Although Mpox cases globally decreased since the end of 2022, numbers are still significant in the African Region, European Region, Region of the Americas, and Western Pacific Region. Rapid and efficient detection of infected individuals by precise screening assays is crucial for successful containment. In these assays, analytical and clinical performance must be assessed to ensure high quality. However, clinical studies evaluating Mpox virus (MPXV) detection kits using patient-derived samples are scarce. This study evaluated the analytical and clinical performance of a new diagnostic MPXV real-time PCR detection kit (Sansure Monkeypox Virus Nucleic Acid Diagnostic Kit) using patient-derived samples collected in Germany during the MPXV clade IIb outbreak in 2022. Our experimental approach determined the Limit of Detection (LoD) to less than 200 cp/mL using whole blood samples and samples derived from vesicles or pustules. Furthermore, we tested potentially inhibiting substances and pathogens with homologous nucleic acid sequences or similar clinical presentation and detected no cross-reactivity or interference. Following this, the assay was compared to a CE-marked test in a clinical performance study and achieved a diagnostic sensitivity of 100.00% and diagnostic specificity of 96.97%. In summary, the investigated real-time PCR assay demonstrates high analytical performance and concurs with the competitor device with high specificity and sensitivity.

**Supplementary Information:**

The online version contains supplementary material available at 10.1007/s00430-024-00800-4.

## Introduction

In recent years, we have been facing a series of epidemic and pandemic outbreaks caused by emerging pathogens such as the SARS-CoV-2 and, more recently, the Mpox virus (MPXV). MPXV, formerly referred to as Monkeypox virus, is an enveloped double-stranded DNA virus classified within the Orthopoxvirus genus. The virus is known to infect a wide range of animal species, including squirrels, rats, dormice, and primates. Moreover, it has the potential for zoonotic transmission to humans, leading to the development of the Mpox disease [1].

Upon infection, individuals typically experience a 3- to 14-day incubation period before the onset of symptoms. Initial symptoms are often non-specific and may include fever, chills, fatigue, headaches, swollen lymph nodes, and the characteristic Mpox rash with highly infectious pustules and vesicles. The virus can spread through direct skin-to-skin contact or exposure to bodily fluids, such as blood, saliva, or sputum [2].

While Mpox has been endemic in some areas of Central and West Africa, a pandemic outbreak starting in 2022 has affected more than 100 countries and resulted in over 95,000 confirmed cases so far [3]. By the end of 2022, the Mpox cases have declined globally, but WHO reports a resurgence in Mpox cases since July 2023, mainly in the African Region, European Region, Region of the Americas, and Western Pacific Region [4].

Effective containment strategies depend on rapid and efficient screening, identification, and isolation of infected individuals. Real-time polymerase chain reaction (PCR) testing is recognized as the gold standard detection method for a wide range of pathogens. However, it necessitates a well-designed assay to ensure high sensitivity and pathogen-specificity.

While commercially available and regulatory-approved MPXV detection kits are limited [5, 6], most PCR protocols have been developed and published as laboratory-developed or laboratory-adapted tests [7–12]. However, these tests may exhibit significant disparities in specificity and sensitivity due to variations in reagents, equipment, and methodologies. Moreover, most of the available PCR protocols and test kits have only been evaluated using recombinant DNA, pseudo-virus particles, or cell-culture-derived MPXV samples, so the specified test parameters may not reflect the actual clinical performance. Furthermore, sample collection, preparation, storage, or handling, as well as variations in the MPXV genome from different patients, may influence the specificity and sensitivity [13, 14].

This emphasizes the need for more readily available, high-quality MPXV diagnostic tools with proven analytical and clinical performance.

In this study, we evaluated the analytical and clinical performance of a new diagnostic MPXV real-time PCR detection kit (Sansure Monkeypox Virus Nucleic Acid Diagnostic Kit). In the analytical performance study, we determined the Limit of Detection (LoD) using various sample types, including whole blood samples or swabs obtained from vesicles or pustules. We assessed cross-reactivity and interference by other pathogens and potentially inhibiting substances. In a clinical performance study, we compared the assay to another CE-marked comparator device using patient-derived samples collected in Germany during the MPXV clade IIb outbreak in 2022.

## Materials and methods

### Specimen collection and preparation

Samples were collected at the Technical University of Munich and the University of Cologne using the Copan Universal Transport Medium (UTM-RT^®^) System or Clinical Virus Transport Medium (Nobel Bioscience, Sinbaek-gil, South Korea). After collection, specimens were stored between − 25 °C and 4 °C.

### Determination of Limit of Detection (LoD)

We used the ATCC Quantitative Synthetic Monkeypox virus DNA (VR-3270SD) to validate the LoD. Absolute quantification of MPXV DNA was determined using the Qiagen QIAcuity Platform.

### DNA isolation

Nucleic acids were extracted using DNeasy Blood & Tissue Kit (Qiagen, Hilden, Germany) or Nucleic Acid Extraction-Purification Kit XCXB (Sansure, Changsha, China) according to the manufacturer’s instructions.

### Real-time PCR

Samples were analyzed for MPXV DNA by real-time PCR on a QuantStudio 5 (ThermoFisher Scientific, Waltham, USA) or a Light cycler 480Z (Roche Diagnostics, Mannheim, Germany) using the Sansure Monkeypox Virus Nucleic Acid Diagnostic Kit (Fluorescence PCR) (Sansure, Changsha, China) or the Bosphore Monkeypox Detection Kit v1 (Anatolia Gene Works, Istanbul, Turkey).

Sansure Monkeypox Virus Nucleic Acid Diagnostic Kit (Fluorescence PCR) is compatible with multiple available PCR instruments and can be used with samples derived from an automated nucleic acid extraction system or a manual extraction. Samples were prepared according to Table 1, and the real-time PCR was performed according to Table 2.

**Table 1:** Sample preparation for the Sansure Monkeypox Virus Nucleic Acid Diagnostic kit (fluorescence PCR)

Reagent	Volume [µL]
MPV PCR/Enzyme Mix	40
Extracted Nucleic Acids	10

**Table 2:** PCR setup for the Sansure Monkeypox Virus Nucleic Acid Diagnostic kit (fluorescence PCR)

Step	Temperature (°C)	Time (s)	Cycles
Decontamination	50	120	1
Polymerase activation	95	5	1
Denaturation	95	5	41
Annealing, Extension, and signal acquisition	60	16	
Device cooling	25	10	1

For the Bosphore Monkeypox Detection Kit v1, samples were prepared according to Table 3, and the real-time PCR was performed according to Table 4.

**Table 3:** Sample preparation for the Bosphore Monkeypox Detection Kit v1

Reagent	Volume [µL]
PCR Master Mix	15
Internal Control	0.2
Extracted Nucleic Acids	5

**Table 4:** PCR setup for the Bosphore Monkeypox Detection Kit v1

Step	Temperature (°C)	Time (s)	Cycles
Initial denaturation	95	600	1
Denaturation	97	20	40
Annealing, Extension, and signal acquisition	60	30	
Device cooling	32	60	1

### Virus culturing

Collected patient samples medium was mixed with 2.5 mL of Dulbecco’s Modified Eagle Medium containing 10% heat-inactivated fetal bovine serum, 1% Penicillin/Streptomycin, and glutamine. Vero E6 cells were seeded into a T-25 flask, inoculated with the sample, and cultured until the cytopathic effect was visible. Once all cells were detached, the supernatant was collected and centrifuged for 10 min at 1,000 x g, the supernatant was discarded, and the pellet was reconstituted in Modified Eagle Medium. The suspension was then subjected to three freeze/thaw cycles (-80 C°/37 C°). Cytopathogenic effects were monitored using an EVOS 5000 microscope (ThermoFisher Scientific, Waltham, USA).

### PCR amplification and sequencing

To verify the initial MPXV DNA diagnostic result, a PCR on extracted supernatants from virus culturing was performed using the Sansure Monkeypox Virus Nucleic Acid Diagnostic Kit (PCR) according to manufacturer instructions, as shown in Tables 5 and 6.

**Table 5:** Sample preparation for the Sansure Monkeypox Virus Nucleic Acid Diagnostic Kit (PCR)

Reagent	Volume [µL]
MPV PCR Mix	40
Extracted Nucleic Acids	10

**Table 6:** Real-time PCR setup for the Sansure Monkeypox Virus Nucleic Acid Diagnostic Kit (PCR)

Step	Temperature (°C)	Time (s)	Cycles
Pre-denaturation	94	300	1
Denaturation	94	20	35
Annealing	59	30	
Extension	72	30	
Final extension	72	600	1

PCR products with a band of approx. 400 bp were gel purified, and Sanger sequenced using the primer MPXV-Seq forward (fw) and reverse (rv) with the sequences shown in Table 7.

**Table 7:** Sequencing primers

Primer	Sequence
MPXV-Seq fw	GTAGTGCTATTGTTTACAGCTCC
MPXV-Seq rv	GCCTTATCGAATACTCTTCCG

## Results

The Sansure Monkeypox Virus Nucleic Acid Diagnostic Kit was evaluated for its analytical and clinical performance with virus isolation, culturing, and sequencing to assess the viral viability of the clinical specimens and to confirm the initial diagnostic results.

### Analytical performance

**Fig. 1:**
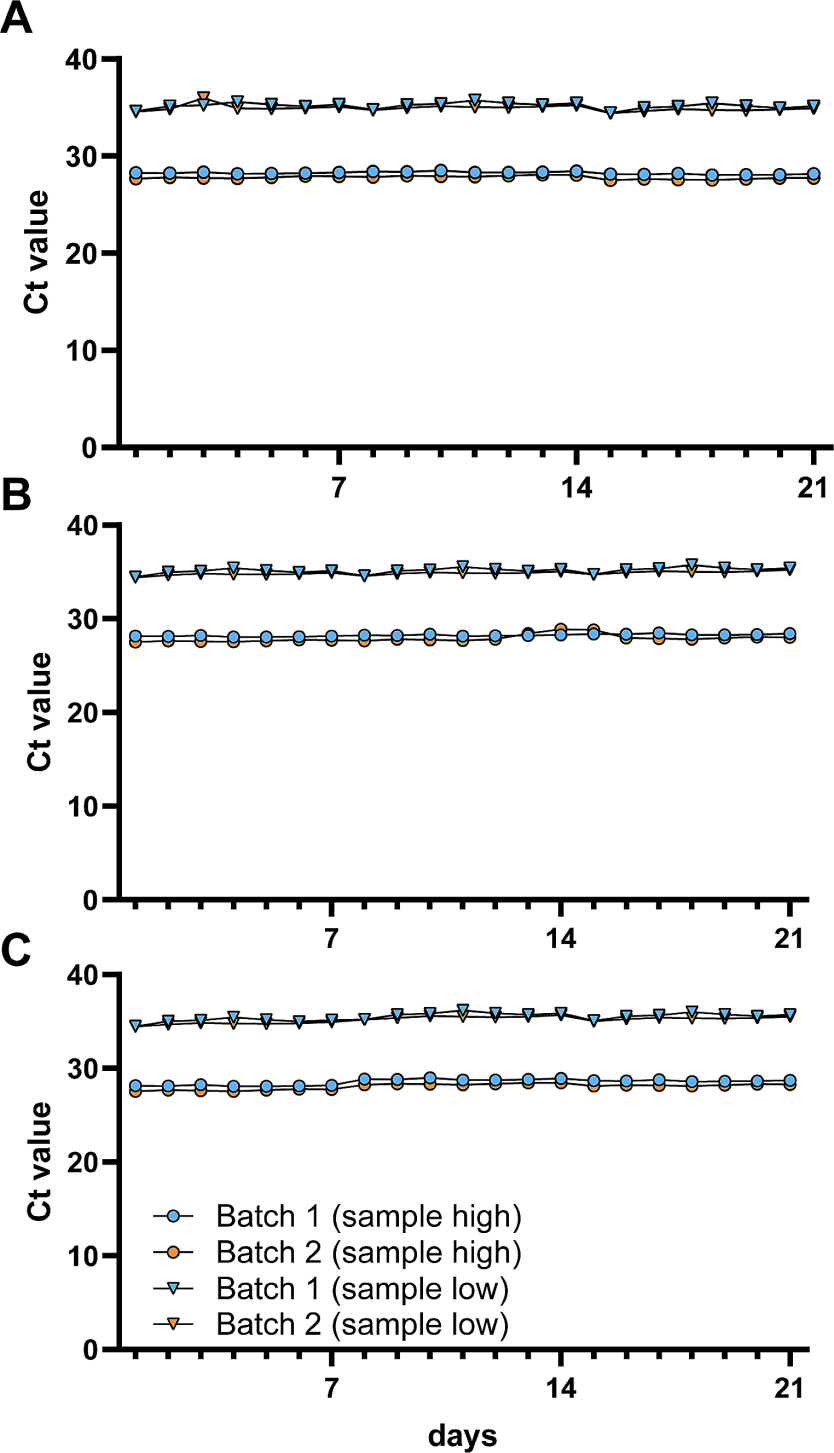
Precision testing in different matrices. MPXV reference DNA was diluted and spiked into MPXV-negative samples of (**A**) whole blood, (**B**) vesicles, and (**C**) pustules and tested over 21 days

For analytical specificity, including specificity-cross reactivity and competitive and endogenous/exogenous interference analysis, we diluted a reference MPXV DNA in either whole blood samples or swaps taken from vesicles or pustules. Each test was conducted with independent extractions to evaluate precision for inter-batch, inter-day, and inter-operator variability. The inter-batch, inter-day, and inter-operator variabilities were all found to be less than 5% (Fig. 1 and Supplementary Tables 1–3).

**Fig. 2:**
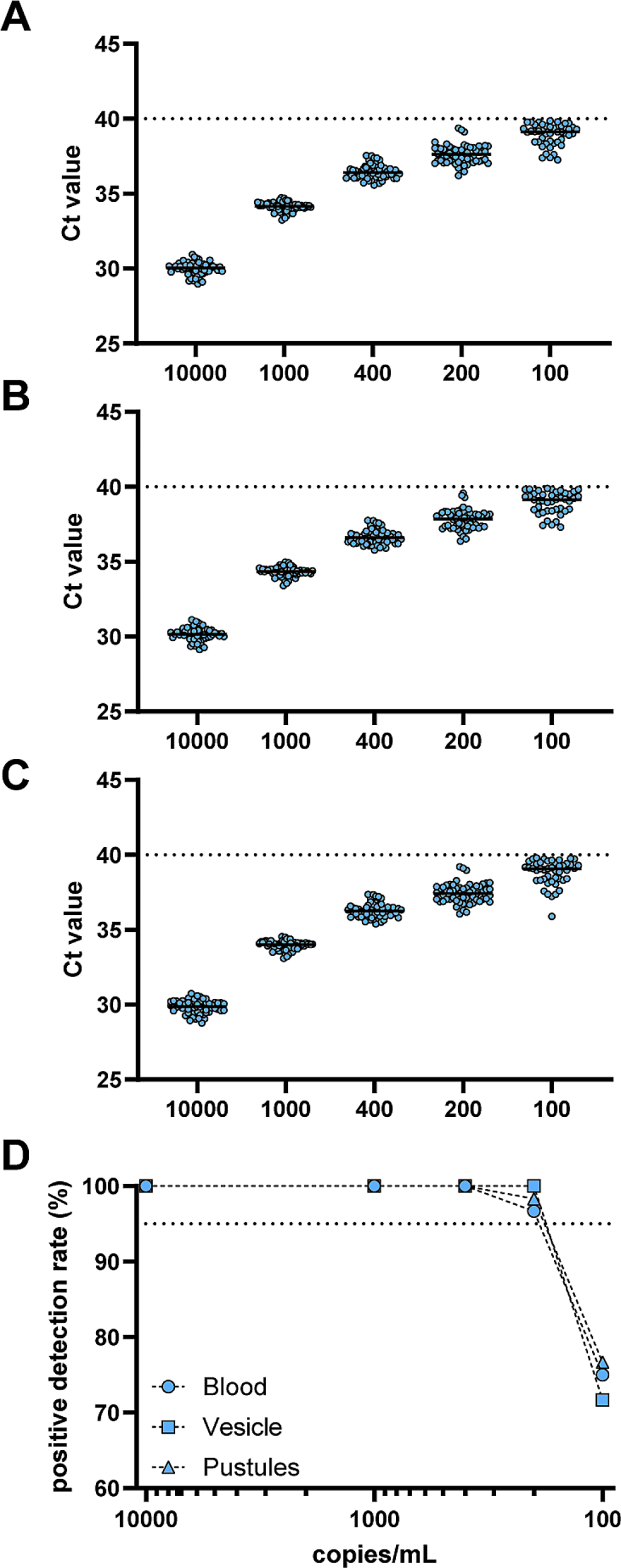
Determination of Limit of Detection (LoD). MPXV-negative samples of whole blood (**A**) or swabs obtained from vesicles (**B**) or pustules (**C**) were inoculated with MPXV DNA. The cycle threshold (Ct) values are presented for varying DNA concentrations spiked into the different matrices. (**D**) The positive detection rate of the MPXV DNA in the different sample types. The dotted line indicates the 95%-positive detection rate

The LoD of the assay was determined by gradually diluting MPXV DNA from 10e4 to 10e2, quantified by dPCR (Fig. 2A-C). Based on these measurements, we determined the LoD for whole blood samples and samples derived from vesicles or pustules as 182 cp/mL, 165 cp/mL, and 119 cp/mL, respectively (Fig. 2D).

Pathogen cross-reactivity was tested using pathogens with homologous nucleic acid sequences, similar clinical presentation, or pathogens frequently present in patients suffering from Mpox. In our analytical evaluation, we tested 19 pathogens in triplicates and could not detect any cross-reactivity (Table 8).

**Table 8:** Analytical cross-reaction testing. All pathogens were tested in triplicates

Pathogen	Positive	Negative
Adenovirus	0 (0%)	3 (100%)
Chlamydia trachomatis	0 (0%)	3 (100%)
Cytomegalovirus	0 (0%)	3 (100%)
Epstein-Barr virus	0 (0%)	3 (100%)
Herpes simplex virus 1	0 (0%)	3 (100%)
Herpes simplex virus 2	0 (0%)	3 (100%)
Human herpes type 6	0 (0%)	3 (100%)
Human herpes type 7	0 (0%)	3 (100%)
Human immunodeficiency virus	0 (0%)	3 (100%)
Measles virus	0 (0%)	3 (100%)
Modified Vaccinia Virus Ankara	0 (0%)	3 (100%)
Mycoplasma genitalum	0 (0%)	3 (100%)
Neisseria gonorrhoeae	0 (0%)	3 (100%)
Parvovirus B19	0 (0%)	3 (100%)
Rotavirus	0 (0%)	3 (100%)
Rubella virus	0 (0%)	3 (100%)
Treponema pallidum	0 (0%)	3 (100%)
Trichomonas vaginalis	0 (0%)	3 (100%)
Varicella zoster virus	0 (0%)	3 (100%)

**Fig. 3:**
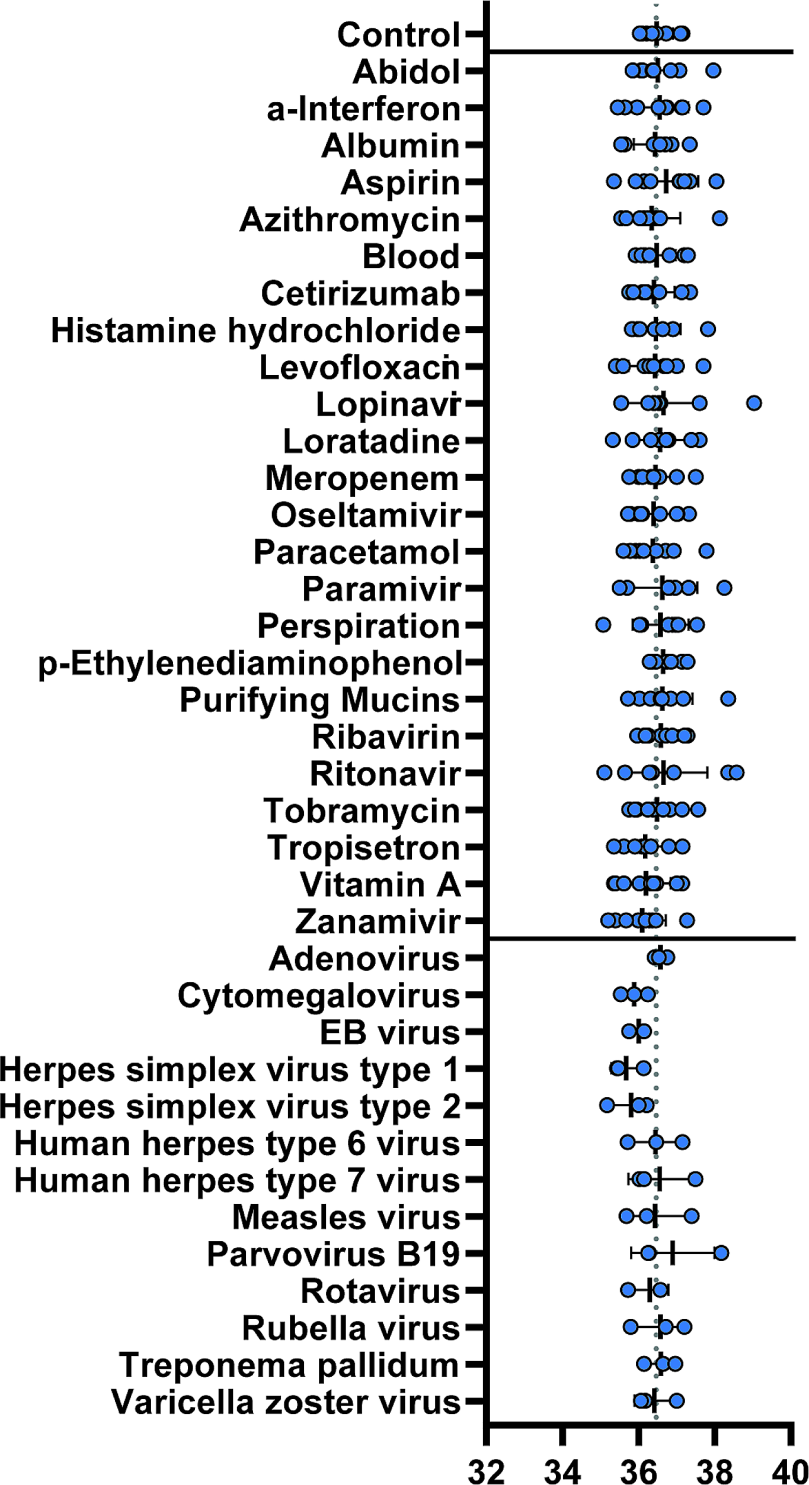
Competitive interference and cross-reactivity testing. Endogenous and exogenous substances were introduced into samples containing a defined MPXV DNA concentration at the LoD. The samples without any additions are labeled as the control (grey). For pathogens, three samples were tested, and for substances, nine samples were tested. The dotted line indicates the mean value of the control. The lines represent the mean, and the whiskers represent the standard deviation (SD)

To assess competitive interference from commonly encountered endogenous or exogenous substances in the sample material, we evaluated the changes in Ct-values by adding different potentially interfering substances (Fig. 3). Detailed information about the concentration used can be found in Supplemental Table 4. None of the tested substances led to a statistically significant change in the Ct value compared to the original control sample.

### Clinical performance

To assess the clinical performance of the assay, we adopted a cross-sectional, observational study design using 63 retrospective samples collected at two large university hospitals in Germany during the MPXV clade IIb outbreak in 2022 with clinical signs and symptoms of a potential infection. The typical symptoms included skin rashes, mucosal lesions, swollen lymph nodes, and fever.

In the initial diagnostic testing, 32 samples tested positive, while 31 tested negative for MPXV. Among the negative samples, there were 21 males and 11 females, with an average age of 51.44 years. Notably, the positive samples did not include females and had a lower average age of 36.74 years, which aligns with the WHO data on Mpox cases of the current outbreak that 96.4% of cases are male and the median age is 34 years [18].

The primary objectives of this study were the diagnostic performance parameters, including sensitivity and specificity. Secondary objectives included predictive values, likelihood ratios, and accuracy.

In the first step, we performed virus culturing followed by PCR amplification and Sanger sequencing to verify the initial diagnostic test results. Notably, all Sanger sequencing results were in accordance with the initial diagnostic results. Subsequently, the Sansure Monkeypox Virus Nucleic Acid Diagnostic Kit (Fluorescence PCR) was compared to another CE-marked comparator device (Bosphore Monkeypox Detection Kit v1). The results of the individual RT-PCRs, including Ct values and test results, are shown in Supplemental Tables 5 and 6. The primary objectives of the two compared devices are shown in Tables 9 and 10. Secondary objectives of the study are shown in Table 11. Both tested kits had high sensitivities and specificities, with the Sansure kit achieving 100% and 96.97%, respectively, and the Bosphore kit reaching 100% and 94.12%, respectively. Further assessed performance parameters in this study included positive and negative predictive values (96.88% and 100%, respectively, for the Sansure kit; 93.94% and 100%, respectively, for the Bosphore kit), and positive and negative likelihood ratios (33.00 and 0.00, respectively, for Sansure kit; 17.00 and 0.00, respectively, for Bosphore kit).

**Table 9:** Test results from the clinical performance of the Bosphore Monkeypox Detection Kit v1

		MPXV DNA confirmed by Sanger sequencing		
		Positive	Negative	Total
Bosphore Kit	Positive	31	2	33
	Negative	0	30	30
	Total	31	32	63

**Table 10:** Test results from the clinical performance of the Sansure Monkeypox Virus Nucleic Acid Diagnostic Kit

		MPXV DNA confirmed by Sanger sequencing		
		Positive	Negative	Total
Sansure Kit	Positive	31	1	32
	Negative	0	31	31
	Total	31	32	63

**Table 11:** Statistical analysis of the clinical performance of each PCR kit

Parameter	Result Bosphore	Result Sansure
Sensitivity	100.00%	100.00%
Specificity	94.12%	96.97%
Positive predictive Value	93.94%	96.88%
Negative predictive Value	100.00%	100.00%
Positive likelihood ratio	17.00	33.00
Negative likelihood ratio	0.00	0.00

In summary, the Sansure Monkeypox Virus Nucleic Acid Diagnostic Kit and the Bosphore Monkeypox Detection Kit v1 demonstrated high sensitivity and specificity. However, the Sansure kit demonstrated slightly superior performance in specificity and positive predictive value.

## Discussion

The containment of infectious diseases, such as Mpox, heavily depends on the availability of reliable in-vitro diagnostic tests. This study evaluated the analytical and clinical performance of the Sansure Monkeypox Virus Nucleic Acid Diagnostic Kit (Fluorescence PCR). Our results show high analytical sensitivity and specificity, reaching 100% in different sample matrixes tested over 21 days and 100% specificity against potentially cross-reacting pathogens. We determined the LoD for MPXV clade IIb to be < 200 cp/mL in different sample matrixes, comparable to other available detection kits [7, 10, 15, 16]. The test sensitivity, at MPXV DNA concentrations close to the calculated LoDs, was not impaired when endogenous or exogenous interfering substances or pathogens were present in the MPXV samples.

While the analytical performance primarily reflects the test´s ability to detect MPXV as a specific analyte, clinical testing is indispensable for assessing sensitivity and specificity in actual patient samples [17]. Thus, we conducted a clinical performance study, including 31 positive and 32 negative retrospective samples collected at two large university hospitals during the MPXV outbreak in Germany. The results of this study revealed that the Sansure Monkeypox Virus Nucleic Acid Diagnostic Kit had a diagnostic sensitivity of 100.00% and diagnostic specificity of 96.97%, achieving a better performance compared to the CE-certified Bosphore Monkeypox Virus Detection Kit with a sensitivity of 100.00% and a specificity of 94,12%, respectively.

Although the sample size might need an extension for a comprehensive evaluation, the results can be used as a performance estimation for statistical sample size calculation for future studies. Considering the current increase in Mpox cases in many world regions, sample collection from other study sites in these regions would be beneficial to cover a broader range of genetic variants of the virus [18, 19].

In conclusion, our results indicate high analytical sensitivity and specificity for the Sansure Monkeypox Virus Nucleic Acid Diagnostic Kit (Fluorescence PCR). The kit concurs in a clinical study with another CE-certified competitor device, the Bosphore Monkeypox Detection Kit v1, with high levels of specificity and sensitivity.

## Electronic supplementary material

Below is the link to the electronic supplementary material.

Supplementary Material 1

## Data Availability

No datasets were generated or analysed during the current study.
