# Supplementary material for: Analytical and clinical evaluation of a novel real-time PCR-based detection kit for Mpox virus
Source: Med Microbiol Immunol. 2024 Aug 5;213(1):18. doi: 10.1007/s00430-024-00800-4 (PMC11300543; doi:10.1007/s00430-024-00800-4)
Supplement: Supplementary file 1 — Supplementary Material 1 [file 430_2024_800_MOESM1_ESM.docx]

**Supplementary Table 1.** Statistical of precision test for whole blood

| **Samples** | | **Medium concentration** | **Borderline concentration** | **Negative** |
| --- | --- | --- | --- | --- |
| Inter-batch | Positive  detection rate | 100.00% | 100.00% | 0.00% |
|  | CV (%) | 0.55% | 0.99% | / |
| Inter-day | Positive  detection rate | 100.00% | 100.00% | 0.00% |
|  | CV (%) | 0.34% | 1.37% | / |
| Inter-operators | Positive  detection rate | 100.00% | 100.00% | 0.00% |
|  | CV (%) | 0.89% | 1.03% | / |

**Supplementary Table 2.** Statistical of precision test for vesicle samples

| **Samples** | | **Medium concentration** | **Borderline concentration** | **Negative** |
| --- | --- | --- | --- | --- |
| Inter-batch | Positive  detection rate | 100.00% | 100.00% | 0.00% |
|  | CV (%) | 0.53% | 0.98% | / |
| Inter-day | Positive  detection rate | 100.00% | 100.00% | 0.00% |
|  | CV (%) | 0.35% | 1.38% | / |
| Inter-operators | Positive  detection rate | 100.00% | 100.00% | 0.00% |
|  | CV (%) | 0.90% | 1.03% | / |

**Supplementary table 3.** Statistical of precision test for pustules samples

| **Samples** | | **Medium concentration** | **Borderline concentration** | **Negative** |
| --- | --- | --- | --- | --- |
| Inter-batch | Positive  detection rate | 100.00% | 100.00% | 0.00% |
|  | CV (%) | 1.11% | 1.27% | / |
| Inter-day | Positive  detection rate | 100.00% | 100.00% | 0.00% |
|  | CV (%) | 0.34% | 1.38% | / |
| Inter-operators | Positive  detection rate | 100.00% | 100.00% | 0.00% |
|  | CV (%) | 0.92% | 1.02% | / |

**Supplementary Table 4.** Concentrations of the tested interfering substances

| **Interfering substance** | **Concentration** |
| --- | --- |
| Purifying Mucins | 20 ug/mL |
| Albumin | 5 g/dL |
| Blood | 5% |
| Perspiration | 5% |
| Paracetamol | 60 ug/mL |
| p-ethylenediaminophenol | 30 ug/mL |
| Aspirin (loanword) | 300 ug/mL |
| Histamine hydrochloride | 200 ug/mL |
| Loratadine | 200 ug/ml |
| Cetirizumab | 20 ug/mL |
| Azithromycin | 100 ug/mL |
| Levofloxacin | 50 ug/mL |
| Tropisetron | 50 ug/mL |
| Meropenem | 50 ug/mL |
| a-interferon | 300 U/mL |
| Zanamivir | 100 ug/mL |
| Ribavirin | 100 ug/mL |
| Oseltamivir | 100 ug/mL |
| Paramivir | 100 ug/mL |
| Lopinavir | 100 ug/mL |
| Ritonavir | 100 ug/mL |
| Abidol | 100 ug/mL |
| Tobramycin | 100 ug/mL |
| Vitamin A | 5 umol/L |

**Supplementary Table 5.** qPCR results of the MPXV positive (confirmed by Sanger sequencing) swab samples.

| **Sample ID** | **Sansure Kit** | | | **Bosphore Kit** | | |
| --- | --- | --- | --- | --- | --- | --- |
|  | **Ct-value**  **MPV (FAM)** | **Ct-value**  **IC**  **(CY5)** | **(MPXV)**  **Result** | **Ct-value**  **MPV (FAM)** | **Ct-value**  **IC**  **(HEX)** | **(MPXV)**  **Result** |
| SNK0001 | 21.60 | 24.07 | Positive | 22.16 | 29.85 | Positive |
| SNK0002 | 30.92 | 28.77 | Positive | 28.73 | 27.53 | Positive |
| SNK0012 | 21.33 | 19.97 | Positive | 19.27 | 30.62 | Positive |
| SNK0014 | 21.93 | 20.07 | Positive | 20.36 | 30.40 | Positive |
| SNK0015 | 21.78 | 19.65 | Positive | 20.26 | 30.41 | Positive |
| SNK0017 | 29.78 | 21.99 | Positive | 28.12 | 28.93 | Positive |
| SNK0018 | 24.63 | 23.81 | Positive | 24.18 | 29.93 | Positive |
| SNK0020 | 20.91 | 19.90 | Positive | 19.56 | 31.37 | Positive |
| SNK0021 | 21.63 | 21.03 | Positive | 21.06 | 29.71 | Positive |
| SNK0022 | 19.75 | 20.10 | Positive | 18.45 | 28.90 | Positive |
| SNK0100 | 30.36 | 29.84 | Positive | 28.77 | 24.97 | Positive |
| SNK0108 | 22.39 | 23.03 | Positive | 22.69 | 26.58 | Positive |
| SNK0111 | 25.96 | 28.02 | Positive | 26.02 | 24.90 | Positive |
| SNK0112 | 21.37 | 21.65 | Positive | 21.97 | 26.15 | Positive |
| SNK0114 | 20.45 | 21.60 | Positive | 20.86 | 26.01 | Positive |
| SNK0116 | 24.96 | 30.80 | Positive | 22.38 | 26.77 | Positive |
| SNK0117 | 21.44 | 22.74 | Positive | 20.47 | 26.74 | Positive |
| SNK0119 | 28.13 | 27.33 | Positive | 27.55 | 25.66 | Positive |
| SNK0122 | 24.44 | 25.77 | Positive | 24.77 | 26.49 | Positive |
| SNK0123 | 27.94 | 32.81 | Positive | 26.31 | 25.25 | Positive |
| SNK0125 | 22.06 | 21.57 | Positive | 21.90 | 26.83 | Positive |
| SNK0134 | 22.38 | 24.27 | Positive | 23.38 | 25.55 | Positive |
| SNK0135 | 21.07 | 31.21 | Positive | 19.20 | 24.98 | Positive |
| SNK0136 | 21.88 | 22.80 | Positive | 22.38 | 26.10 | Positive |
| SNK0137 | 23.69 | 38.02 | Positive | 22.93 | 25.73 | Positive |
| SNK0138 | 31.31 | 32.47 | Positive | 28.45 | 25.53 | Positive |
| SNK0139 | 32.22 | 32.28 | Positive | 29.88 | 25.64 | Positive |
| SNK0140 | 31.04 | 32.09 | Positive | 32.19 | 25.91 | Positive |
| SNK0141 | 27.82 | 31.55 | Positive | 25.60 | 25.97 | Positive |
| SNK0142 | 25.79 | 28.12 | Positive | 25.73 | 26.15 | Positive |
| SNK0145 | 31.89 | 30.36 | Positive | 32.01 | 26.49 | Positive |

**Supplementary Table 6.** Real-time PCR results of the MPXV negative (confirmed by Sanger sequencing) swab samples.

| **Sample ID** | **Sansure Kit** | | | **Bosphore Kit** | | |
| --- | --- | --- | --- | --- | --- | --- |
|  | **Ct-value**  **MPV (FAM)** | **Ct-value**  **IC**  **(CY5)** | **(MPXV)**  **Result** | **Ct-value**  **MPV (FAM)** | **Ct-value**  **IC**  **(HEX)** | **(MPXV)**  **Result** |
| SNK0003 | 0 | 22.84 | Negative | 0 | 29.11 | Negative |
| SNK0004 | 0 | 21.15 | Negative | 0 | 28.83 | Negative |
| SNK0005 | 38.96 | 22.34 | Positive | 0 | 28.98 | Negative |
| SNK0006 | 0 | 23.98 | Negative | 36.34 | 31.67 | Positive |
| SNK0007 | 0 | 28.21 | Negative | 36.56 | 31.99 | Positive |
| SNK0011 | 0 | 28.26 | Negative | 0 | 29.58 | Negative |
| SNK0013 | 0 | 28.42 | Negative | 0 | 29.23 | Negative |
| SNK0016 | 0 | 25.52 | Negative | 0 | 28.50 | Negative |
| SNK0019 | 0 | 22.88 | Negative | 0 | 28.58 | Negative |
| SNK0023 | 0 | 25.60 | Negative | 0 | 28.86 | Negative |
| SNK0024 | 0 | 24.36 | Negative | 0 | 28.00 | Negative |
| SNK0025 | 0 | 26.65 | Negative | 0 | 28.92 | Negative |
| SNK0070 | 0 | 23.95 | Negative | 0 | 24.84 | Negative |
| SNK0071 | 0 | 33.22 | Negative | 0 | 24.80 | Negative |
| SNK0072 | 0 | 37.35 | Negative | 0 | 25.10 | Negative |
| SNK0073 | 0 | 21.95 | Negative | 0 | 24.50 | Negative |
| SNK0074 | 0 | 32.72 | Negative | 0 | 24.85 | Negative |
| SNK0075 | 0 | 20.64 | Negative | 0 | 25.28 | Negative |
| SNK0076 | 0 | 26.78 | Negative | 0 | 25.19 | Negative |
| SNK0077 | 0 | 24.87 | Negative | 0 | 24.94 | Negative |
| SNK0078 | 0 | 23.68 | Negative | 0 | 25.39 | Negative |
| SNK0079 | 0 | 25.06 | Negative | 0 | 24.91 | Negative |
| SNK0080 | 0 | 24.69 | Negative | 0 | 25.37 | Negative |
| SNK0081 | 0 | 25.98 | Negative | 0 | 24.41 | Negative |
| SNK0082 | 0 | 20.11 | Negative | 0 | 24.79 | Negative |
| SNK0085 | 0 | 21.20 | Negative | 0 | 25.93 | Negative |
| SNK0086 | 0 | 39.01 | Negative | 0 | 25.69 | Negative |
| SNK0087 | 0 | 27.71 | Negative | 0 | 26.51 | Negative |
| SNK0088 | 0 | 38.48 | Negative | 0 | 26.28 | Negative |
| SNK0089 | 0 | 23.02 | Negative | 0 | 25.43 | Negative |
| SNK0090 | 0 | 32.18 | Negative | 0 | 26.13 | Negative |
| SNK0091 | 0 | 27.31 | Negative | 0 | 26.54 | Negative |
